# Impact of Virtual Rehabilitation in Adjunct to Conventional Physical Therapy on Proximal Humerus Fracture: A Randomized Controlled Trial

**DOI:** 10.7759/cureus.56022

**Published:** 2024-03-12

**Authors:** Abhishek Daf, Deepali S Patil

**Affiliations:** 1 Department of Musculoskeletal Physiotherapy, Ravi Nair Physiotherapy College, Datta Meghe Institute of Higher Education and Research, Wardha, IND

**Keywords:** vr-based training, physiotherapy management, upper limb function, shoulder dysfunction, shoulder impairments

## Abstract

Introduction

Humerus fractures are fairly prevalent in the general population, and their incidence increases with age. The majority of minimally displaced fractures may be treated with prompt rehabilitation. An interdisciplinary team strategy involving experienced musculoskeletal specialists, geriatricians, and skilled physiotherapists should be employed for optimal therapy. Rehabilitation is essential in coping with the consequences of the fracture. The greatest current information for shoulder rehabilitation comprises the use of counseling, exercises, and mobility of restricted joints to regain upper limb function. Virtual reality (VR) based therapies are among the most effective since they may give visual, aural, and somatosensory cues to help patients. In fact, VR-based treatments have been shown to enhance motor function, neuropathic pain, balance, and aerobic performance in individuals with neurological disorders. However, there is limited evidence on the use of VR’s therapeutic efficacy on individuals with musculoskeletal disorders. This study applied oculus-guided VR rehabilitation in addition to conventional physical therapy for the rehabilitation of patients with proximal humerus fractures. This study aims to assess the impact of virtual rehabilitation in adjunct to conventional physical therapy on proximal humerus fracture.

Methods

This study is a randomized controlled trial in which 50 patients were divided randomly into two groups: 25 patients in group A and 25 patients in group B. Group A was an experimental group that received VR plus conventional therapy. At the same time, group B was a control group that received only conventional therapy. Shoulder range of motion (ROM), manual muscle testing (MMT), numerical pain rating scale (NPRS), disabilities of arm, shoulder, and hand (DASH), and Shoulder pain and disability index (SPADI) were used as outcome measures of this study.

Results

There is an increase in flexion (t=7.58, P=0.0001), extension (t=6.90, P=0.0001), abduction (t = 9.57, P=0.0001), internal rotation (t=6.31, P=0.0001), and external rotation (t=3.41, P=0.001) in group A is statistically more significant than group B. The improvement in MMT scores in group B (t=1.71, P=0.10) is not significant, whereas improvements in group A are statistically significant (t=13.86, P=0.0001). The SPADI and DASH scores improved more significantly in group A (t=62.46, P=0.0001, and t=57.48, P=0.0001, respectively) than in group B (t=39.14, P=0.0001 and 46.58, P=0.0001, respectively). There is no significant difference in pain outcomes between the two groups.

Conclusion

The findings of this study reveal that virtual rehabilitation in adjunct to conventional physical therapy on proximal humerus fracture is more effective in improving shoulder ROM, muscle strength, and upper limb function than conventional therapy alone. However, no intervention can be considered superior to others in terms of the management of pain associated with proximal humerus fracture.

## Introduction

Humerus fractures are fairly prevalent in the general population. These fractures make up about 7% to 8% of all fractures occurring in elderly individuals from Western countries. The frequency of these fractures increases with the advancing age [1]. In Western countries, proximal humerus fractures (PHF) make up approximately 6% of all fractures. The majority of PHF with small displacement of fragments can be treated with prompt rehabilitation. For displaced fractures, percutaneous fixation, intramedullary nailing, interlocking plating, and replacement are all preferred choices of management [2]. To produce the desired output, a thorough fracture assessment, careful patient monitoring with careful evaluation of presenting features, pathologies, and functional demands, and effective surgical expertise all over a diverse variety of reconstruction surgery and arthroplasty alternatives are required. An interdisciplinary team strategy involving experienced musculoskeletal specialists, geriatricians, and skilled physiotherapists should be employed for optimal therapy [3].

For years after the incident, many continue to have shoulder issues as a consequence of the fracture. Rehabilitation is essential in coping with the consequences of the fracture. The greatest current information for shoulder rehabilitation comprises the use of counseling, exercises, and mobility of restricted joints to regain upper limb function [4]. Patients with upper limb deficits should often be trained to use appropriate therapeutic equipment continuously to recover. Exercises skates for the hand and arm, a vertical tower, an inclination board, stacking cones, and mobility exercises are among the most regularly used equipment items. Passive or patient education through structural components, video games that allow patients to comply with the advice depicted on the screen to move exoskeletons to aid neurological restoration, and VR that combines and enhances audio, video, visual features, and notifications to begin making people believe they are experiencing it for actual are just a few illustrations of therapeutic approaches [5]. The healing period for most injuries and deficits is lengthy, difficult, and boring; as a consequence, sufferers may lack the ambition to stick to physical therapy, resulting in poor recovery and progress efficiency. As an outcome, new tactics must be developed to improve the frequency, time, intensity, inspiration, and enjoyment of the exercises so that patients can undertake them at home [6].

Virtual reality (VR) is a revolutionary technique that has gained ground in the medical sector in recent years, and it is being used to treat a wide spectrum of illnesses. VR technology uses computers to replicate the external world. The user can interact with this simulated world by using hand-held controllers. The visual feedback allows the patients to correct their movements and thus facilitate improvements in their motor function [7]. Virtual worlds and tasks may be adapted to the participant's cognitive and physical limitations, which is important for restructuring and reactivating those brain regions associated with motor planning, training, and performance, as well as sustaining interest [8].

VR is among the most effective therapies, since it may give visual, aural, and somatosensory cues that help to improve the gait of Parkinson's disease (PD) patients. It allows users to interact with a virtual environment while health experts track and assess their development. External signals help patients with PD improve their stride, and the use of visual information leads to an extra gain in speed. Thus, this modality can be applied in conjunction with already proven conventional therapies [9]. Several recent systematic evaluations on the application of VR training in lower limb stroke recovery recommend the use of VR for balance and ambulation training. VR has also been proven to enhance upper limb motor performance in persons who have had a stroke and have persistent hemiparesis [10]. VR-based therapies have been shown to enhance motor function, neuropathic pain, balance, and aerobic capacity in individuals with spinal cord injuries [11].

However, there is currently no proof of VR's therapeutic efficacy on individuals with musculoskeletal disorders. In addition, research has indicated that VR can help with pain control, such as pain reduction during burn victim bandage changes. VR can also help to lower anxiety, divert from pain fears, and relieve tension. It has the potential to distract the attention of individuals who are frightened to move due to discomfort [12]. The Oculus Quest is a headgear that covers the eyes and provides a 6 degrees of freedom experience. It was designed with intensive gaming in mind. The user's field of view is completely obscured; each motion of the neck is evaluated, and the interaction is thoroughly engrossing. Because the advantages of VR distraction grow with immersion, it is feasible that VR will be much more effective at diverting from physical and psychological suffering [13].

## Materials and methods

Materials

Numerical pain rating scale (NPRS), universal goniometer, marker, Shoulder Pain and Disability Index (SPADI), Disabilities of the arm, shoulder, and hand questionnaire (DASH), oculus Quest, shoulder pulley, shoulder wheel, finger ladder, couch, resistance bands, dumbbells, squeeze ball, and wrist and finger exerciser.

Procedure

This randomized controlled trial (RCT) was conducted at Ravi Nair Physiotherapy College, Sawangi (Meghe), Wardha, India after obtaining ethical clearance from the Institutional Ethical Committee (IEC). The duration of the study was six months from September 2022 to February 2023. The study took place This RCT included 50 participants with PHFs with the inclusion criteria that both male and female patients of age between 40 and 60 years, managed operatively using a Buttress plate and Intramedullary nailing. Exclusion criteria included patients with any diagnosed psychiatric illness, history of brain trauma, visual and auditory defects, severe cognitive impairments, CNS or PNS involvement, fractures other than PHF, and shoulder pain due to other causes.

**Figure 1:**
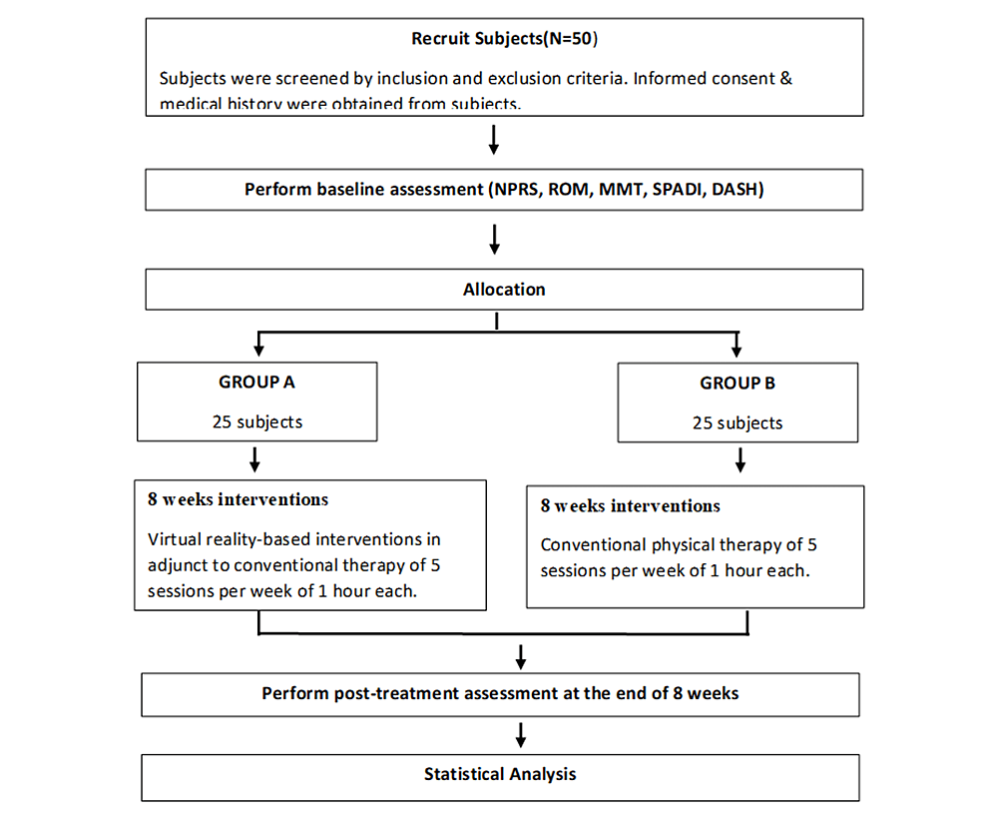
Flowchart of the study procedure NPRS: Numerical Pain Rating Scale, ROM: Range of Motion, MMT: Manual Muscle Testing, SPADI: Shoulder Pain and Disability Index, DASH: Disabilities of Arm, Shoulder and Hand

The patients who met the eligibility criteria were informed about necessary details related to this study. Then all the patients were asked to give their written informed consent. The willing participants were assessed for baseline NPRS, ROM, MMT, SPADI, and DASH questionnaires. The Simple Random Sampling Technique (concealed envelope) was used to randomly allocate the participants to Group A and Group B. Each group had 25 participants. The participants in each group received respective interventions for eight weeks and the outcome measure scores were obtained at the end of eight weeks. Then, pre- and post-outcome measure scores were compared and analyzed statistically. Figure 1 shows the summary of the procedure.

Interventions

Group A

**Figure 2:**
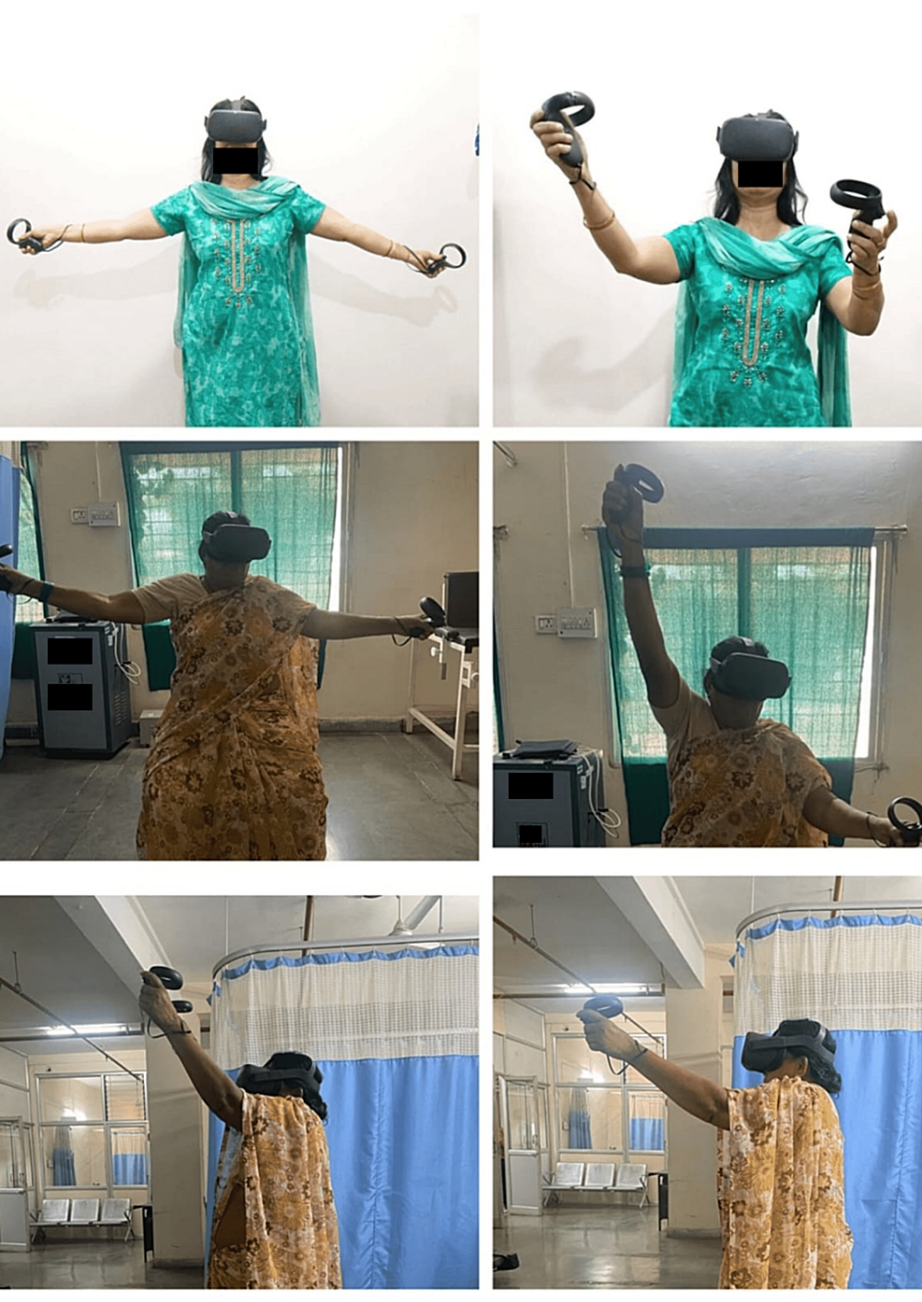
Patients performing VR-based upper limb activities using an Oculus headset and handheld controller. VR: Virtual Reality

Group A was the interventional group, receiving a combination of VR-based exercises and conventional physical therapy (PT) for eight weeks. The duration of each session was 60 minutes which included 30 minutes of VR-based exercises and 30 minutes of conventional PT interventions. are performed for 30 minutes/day, five days/week. All the interventions were given for five days/week. VR-based exercises were delivered using VR games like skiing, flip-flop, boxing, and kitchen simulation. The games consist of simulated environments that facilitate the movements of the upper limbs as shown in Figure 2. Lift the affected extremity to a specified height and then hold the position for five seconds to facilitate the shoulder ROM activity, eating an apple using the affected hand to mimic elbow curls, grip an imagery object firmly to facilitate grip strengthening exercises, reaching out to the objects at different height with affected upper extremity, and hit an object in front with a firm grip. Imitate a dummy in a video performing upper limb ROM exercises. Conventional PT included mobilization technique (Maitland mobilization), resisted isometrics, scapular sets, shoulder ROM exercises, elbow curls, wrist strengthening exercises, and handgrip strengthening exercises.

Group B

**Figure 3:**
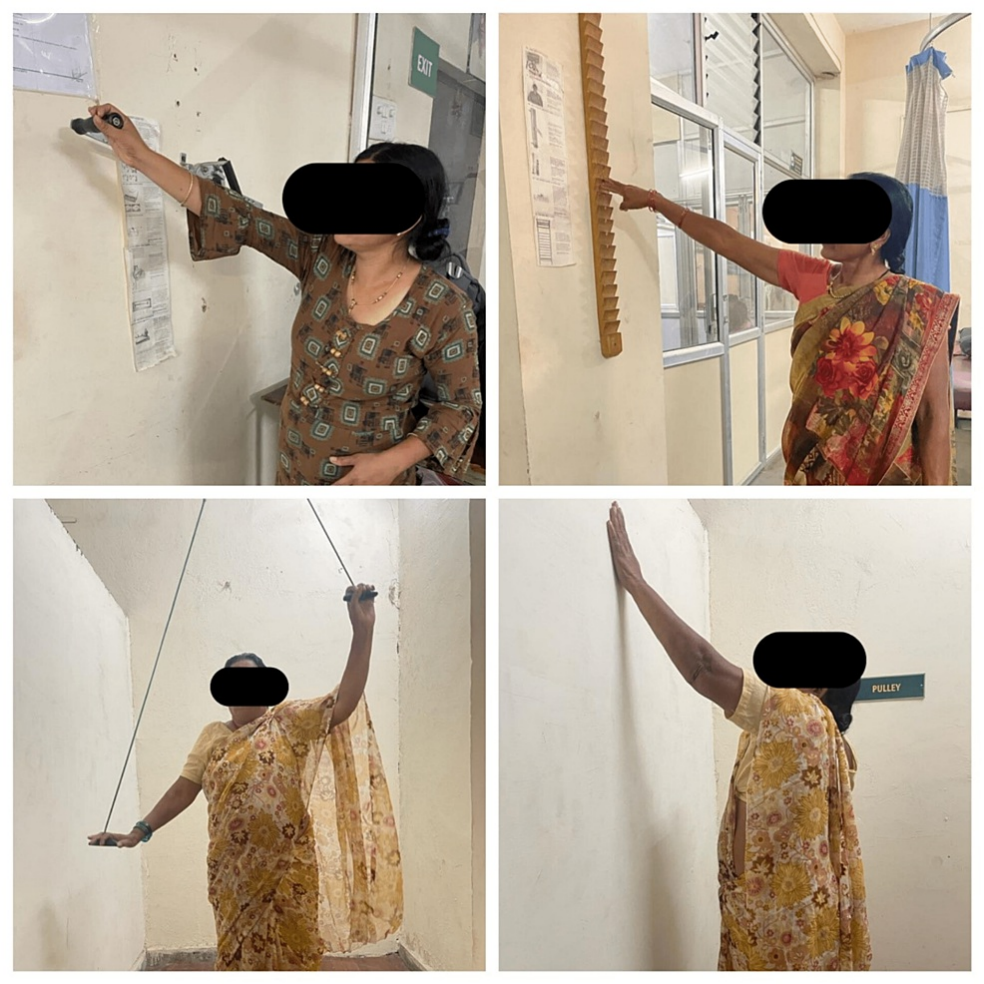
Patients performing conventional exercises like shoulder pully exercise, finger ladder exercise, shoulder wheel exercises, and wall washing exercises.

Group B was the control group, with subjects receiving only conventional PT for eight weeks. The duration of each session was one hour with the frequency of five sessions/week. The conventional PT interventions included mobilization technique, isometrics, scapular sets, shoulder ROM exercises as permitted, elbow curls, wrist strengthening exercises, and hand grip strengthening exercises. ROM exercises included pendular exercises, wall washing exercises, tabletop polishing exercises, cradle exercises, finger ladder exercises, shoulder pulley exercises, and shoulder wheel exercises as shown in Figure 3. Strengthening exercises using dumbbells, free weights, and resistance bands. Grip strengthening exercises using squeeze ball, and wrist and finger exerciser.

## Results

Statistical analysis was done using SPSS 27.0 version software (IBM Corp., Armonk, NY). Descriptive and inferential data were statistically analyzed using student’s paired and unpaired t-tests for within-group and between-group comparison respectively. P <0.05 is considered the significance level.

**Figure 4:**
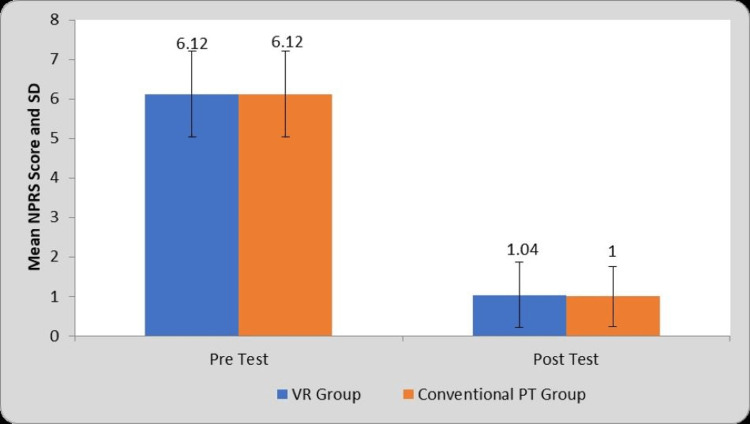
Graphical representation of a comparison of NPRS Score in two groups at pre- and post-test. VR: Virtual Reality, PT: Physiotherapy, NPRS: Numerical Pain Rating Scale, SD: Standard Deviation

Table 1 and Figure 4 depict the within-group and between-group comparisons of NPRS scores before and after the interventions. The mean NPRS scores for group A (VR group) are 6.12±1.09 and 1.04±0.73 on the pre-test and post-test respectively. The mean NPRS scores for group B (Conventional PT group) are 6.12±1.09 and 1±0.76 at the pre-test and post-test respectively. The within-group comparison of pre- and post-NPRS scores shows significant pain relief in both groups (P=0.0001). However, a between-group comparison shows no significant difference in pain outcome at post-test.

**Table 1:** Comparison of NPRS Score in two groups at pre- and post-test. VR: Virtual Reality, PT: Physiotherapy, NPRS: Numerical Pain Rating Scale, S: Significant, NS: Not significant, MD: Mean Difference

Group	Pre-test	Post-test	MD	Student’s paired t-test value
VR Group	6.12±1.09	1.04±0.73	5.08±0.57	44.44, P=0.0001, S
Conventional PT Group	6.12±1.09	1±0.76	5.12±0.52	48.67, P=0.0001, S
Student’s unpaired t-test				
t-value	0.00, P=1.00, NS	0.18 P=0.85, NS		

**Figure 5:**
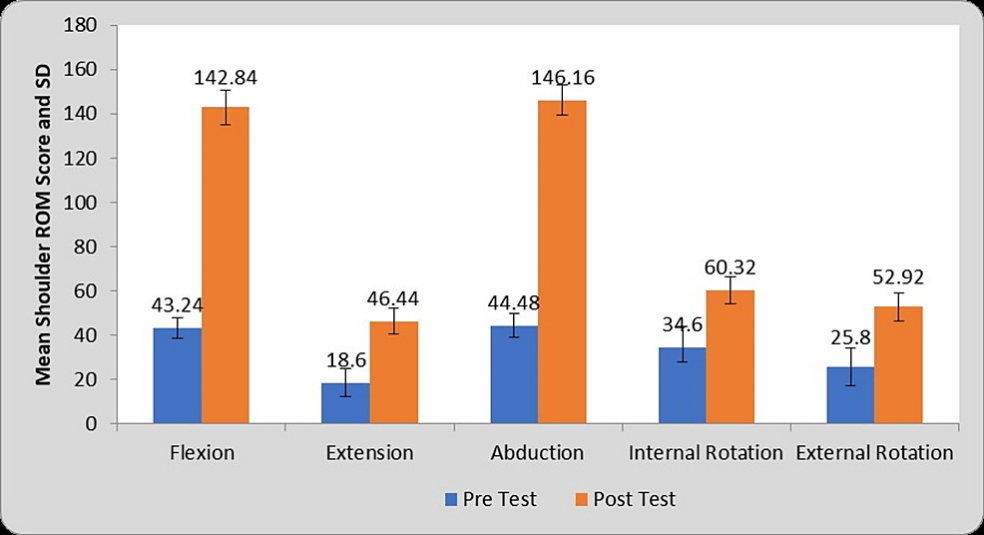
Graphical representation of comparison of shoulder ROM values in VR Group (Group A) at pre- and post-test. VR: Virtual Reality, ROM: Range of Motion, SD: Standard Deviation

Table 2 and Figure 5 depict the comparison between pre- and post-test shoulder ROM values in the VR group (Group A). For group A (VR group) the mean difference values of flexion (99.60±7.05), extension (27.84±5.55), abduction (101.68±8.27), internal rotation (25.72±7.13), external rotation (27.12±5.42) ROMs after statistical comparison of mean ROM values of pre- and post-test reveals significant increase in all the shoulder ROM values (P=0.0001).

**Table 2:** Comparison of Shoulder ROM Score in VR Group (Group A) at pre- and post-test. VR: Virtual Reality, ROM: Range of Motion, S: Significant, MD: Mean Difference

Shoulder ROM	Pre-test	Post-test	MD	Student’s paired t-test value
Flexion	43.24±4.74	142.84±7.88	99.60±7.05	70.45, P=0.0001, S
Extension	18.60±6.21	46.44±5.96	27.84±5.55	25.07, P=0.0001, S
Abduction	44.48±5.27	146.16±6.94	101.68±8.27	61.43, P=0.0001, S
Internal Rotation	34.60±9.45	60.32±6.12	25.72±7.13	18.02, P=0.0001, S
External Rotation	25.80±8.37	52.92±6.37	27.12±5.42	24.99, P=0.0001, S

**Figure 6:**
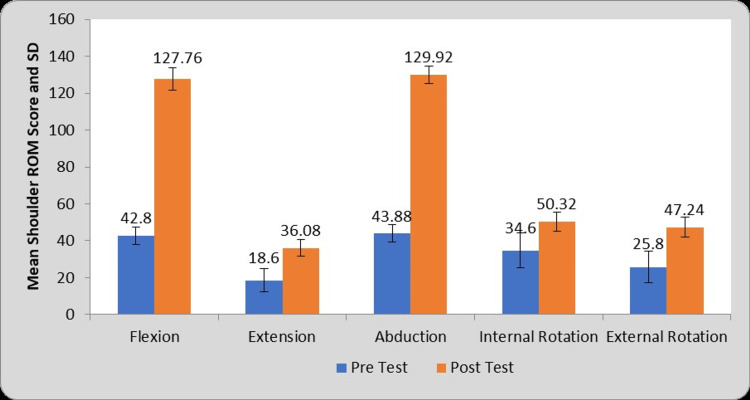
Graphical representation of a comparison of shoulder ROM score in conventional PT group (Group B) at pre- and post-test. PT: Physiotherapy, ROM: Range of Motion, SD: Standard Deviation

Table 3 and Figure 6 depict the comparison between pre- and post-treatment shoulder ROM values in group B. For group B (Conventional PT group) the mean difference values of flexion (84.96±7.62), extension (17.48±5.30), abduction (86.04±5.76), internal rotation (15.72±8.34), external rotation (21.44±6.36) ROMs after statistical comparison of mean ROM values of pre- and post-test reveals significant improvements in all the shoulder ROM values (P=0.0001).

**Table 3:** Comparison of shoulder ROM score in conventional PT group (Group B) at pre- and post-test. PT: Physiotherapy, ROM: Range of Motion, S: Significant, MD: Mean Difference

Shoulder ROM	Pre-test	Post-test	MD	Student’s paired t test t-value
Flexion	42.80±4.65	127.76±6.04	84.96±7.62	55.68, P=0.0001, S
Extension	18.60±6.21	36.08±4.54	17.48±5.30	16.46, P=0.0001, S
Abduction	43.88±4.72	129.92±4.86	86.04±5.76	74.56, P=0.0001, S
Internal Rotation	34.60±9.45	50.32±5.01	15.72±8.34	9.41, P=0.0001, S
External Rotation	25.80±8.37	47.24±5.35	21.44±6.36	16.84, P=0.0001, S

**Figure 7:**
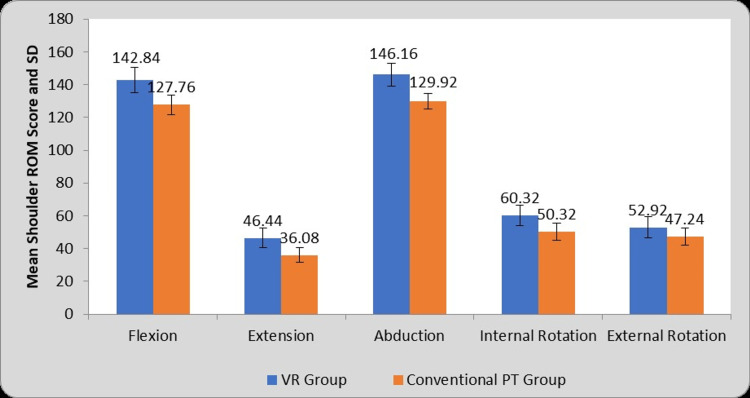
Graphical representation of comparison of Shoulder ROM Score in VR (Group A) and Conventional PT (Group B) Groups at post-test. VR: Virtual Reality, PT: Physiotherapy, ROM: Range of Motion, SD: Standard Deviation

Table 4 and Figure 7 depict the comparison of shoulder ROM values between VR (Group A) and conventional PT (Group B) Groups at post-test assessment. Comparison of post-treatment mean shoulder ROM values between group A and group B gives the mean difference in flexion (15.08±1.98), extension (10.36±1.50), abduction (16.24±1.69), internal rotation (10±1.58), external rotation (5.68±1.66). There are significant improvements in all the shoulder ROMs in group A than in group B at post-test assessment (P=0.0001). 

**Table 4:** Comparison of Shoulder ROM Score in VR (Group A) and Conventional PT (Group B) Groups at post-test. VR: Virtual Reality, PT: Physiotherapy, ROM: Range of Motion, S: Significant, MD: Mean Difference

Shoulder ROM	VR Group	Conventional PT Group	MD	Student’s unpaired t-test value
Flexion	142.84±7.88	127.76±6.04	15.08±1.98	7.58, P=0.0001, S
Extension	46.44±5.96	36.08±4.54	10.36±1.50	6.90, P=0.0001, S
Abduction	146.16±6.94	129.92±4.86	16.24±1.69	9.57, P=0.0001, S
Internal Rotation	60.32±6.12	50.32±5.01	10±1.58	6.31, P=0.0001, S
External Rotation	52.92±6.37	47.24±5.35	5.68±1.66	3.41, P=0.001, S

**Figure 8:**
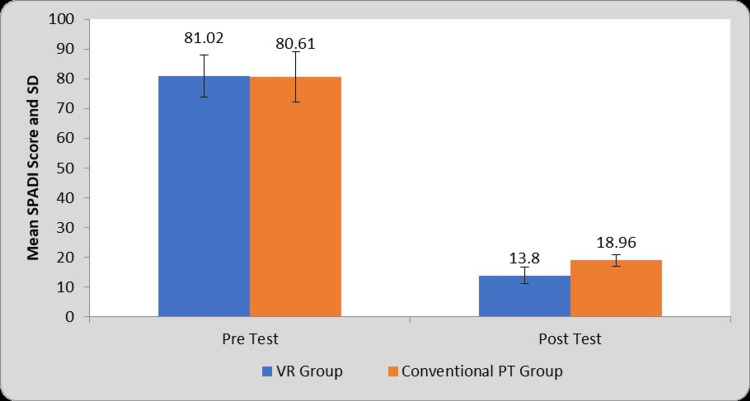
Graphical representation of a comparison of SPADI score in two groups at pre- and post-test. VR: Virtual Reality, PT: Physiotherapy, SPADI: Shoulder pain and disability index, SD: Standard Deviation

Table 5 and Figure 8 depict within-group and between-group comparisons of mean SPADI scores before and after the interventions. Statistical analysis obtained the mean difference of 67.22±5.38 and 61.65±7.87 in group A and group B respectively. There is a significant increase in SPADI scores in both groups. However, results also show a significant difference in post-treatment SPADI scores between the two groups in favor of group A (P=0.0001).

**Table 5:** Comparison of SPADI Score in two groups at pre- and post-test. VR: Virtual Reality, PT: Physiotherapy, SPADI: Shoulder pain and disability index, S: Significant, NS: Not significant, MD: Mean Difference

Group	Pre-test	Post-test	MD	Student’s paired t-test value
VR Group	81.02±7.07	13.80±2.79	67.22±5.38	62.46, P=0.0001, S
Conventional PT Group	80.61±8.39	18.96±1.89	61.65±7.87	39.14, P=0.0001, S
Student’s unpaired t-test				
t-value	0.18, P=0.85, NS	7.62, P=0.0001, S	-	-

**Figure 9:**
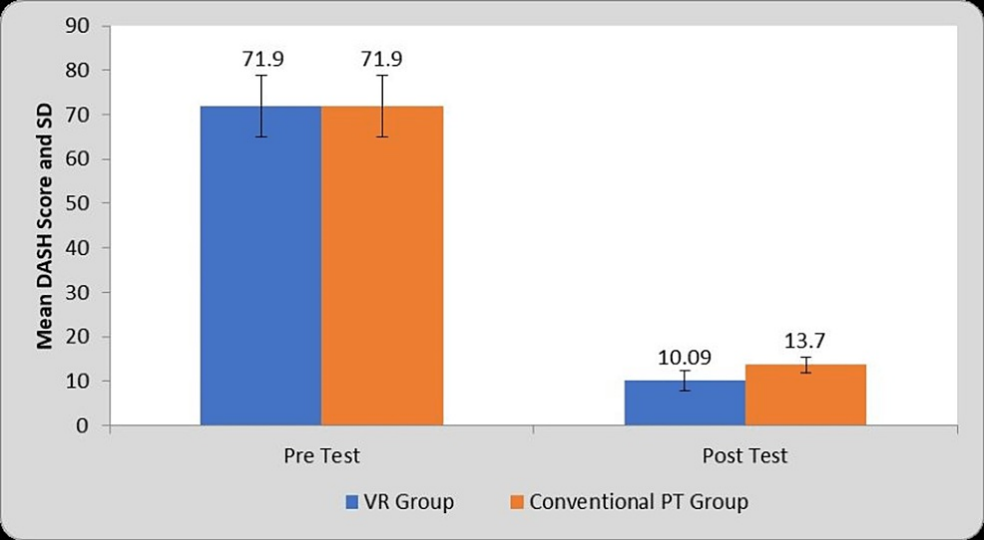
Graphical representation of comparison of DASH score in two groups at pre- and post-test. VR: Virtual Reality, PT: Physiotherapy, DASH: Disability of Arm, Shoulder and Hand, SD: Standard Deviation

Table 6 and Figure 9 depict the within-group and between-group comparison of mean DASH scores before and after the interventions. Statistical analysis obtained the mean difference of 61.81±5.37 in group A and 58.20±6.24 in group B. There is a significant increase in DASH scores in both groups. However, results also show a significant difference in post-treatment DASH scores between the two groups in favor of group A (P=0.0001).

**Table 6:** Comparison of DASH Score in two groups at pre- and post-test. VR: Virtual Reality, PT: Physiotherapy, DASH: Disability of Arm, Shoulder and Hand, S: Significant, NS: Not significant, MD: Mean Difference

Group	Pre-test	Post-test	MD	Student’s paired t-test value
VR Group	71.90±6.87	10.09±2.27	61.81±5.37	57.48, P=0.0001, S
Conventional PT Group	71.90±6.87	13.70±1.75	58.20±6.24	46.58, P=0.0001, S
Student’s unpaired t-test				
t-value	0.00, P=1.00, NS	6.29, P=0.0001, S	-	-

**Figure 10:**
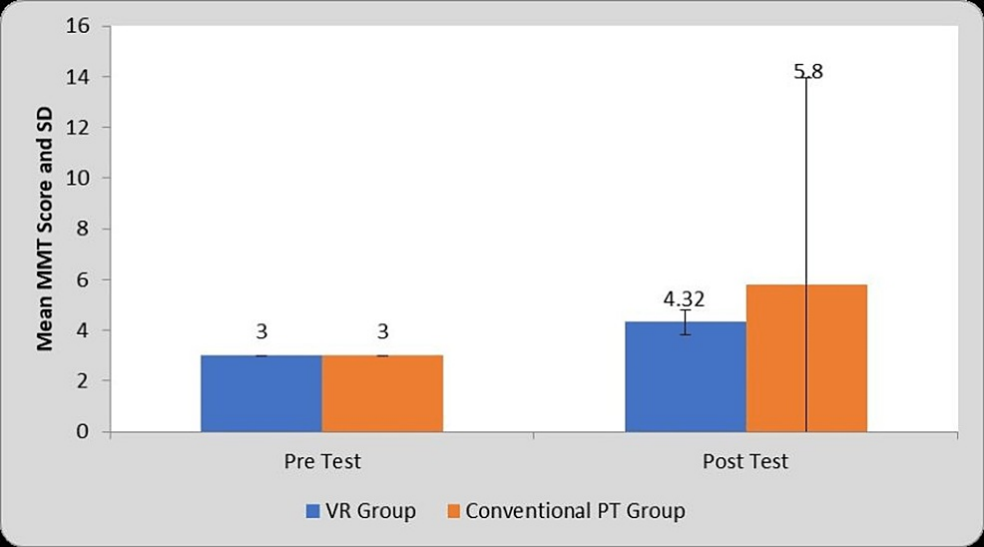
Graphical representation of comparison of MMT Score in two groups at pre- and post-test. VR: Virtual Reality, PT: Physiotherapy, MMT: Manual Muscle Testing, SD: Standard Deviation

Table 7 and Figure 10 depict within-group and between-group comparative analysis of mean DASH scores before and after the interventions. Statistical comparison of pre- and post-treatment mean MMT scores obtained the mean difference of 1.32±0.47 and 2.80±8.17 in group A and group B respectively. The improvement in MMT scores in group B (t=1.71, P=0.10) is not significant whereas improvements in group A are statistically significant (t=13.86, P=0.0001).

**Table 7:** Comparison of MMT Score in two groups at pre- and post-test. VR: Virtual Reality, PT: Physiotherapy, MMT: Manual Muscle Testing, S: Significant, NS: Not significant, MD: Mean Difference

Group	Pre-test	Post-test	MD	Student’s paired t test t-value
VR Group	3±0	4.32±0.47	1.32±0.47	13.86, P=0.0001, S
Conventional PT Group	3±0	5.80±8.17	2.80±8.17	1.71, P=0.10, NS
Student’s unpaired t-test				
t-value	-	0.90, P=0.37, NS	-	-

Statistical analysis of pre- and post-test scores shows significant improvements in ROM, MMT, NPRS, and disability scores in both groups. However, statistical between-group comparison reveals significant improvements in group A than in group B. This demonstrates that VR-based exercises in adjunct to conventional physical therapy exercises are more beneficial than conventional physical therapy only.

## Discussion

This RCT aimed to study the impact of VR-based exercises in adjunct to conventional PT in patients who sustained PHF and then managed operatively with buttress plate and intramedullary nailing. For this purpose, the participants were randomly allocated into two groups, 25 participants in each group. Group A received a combination of VR-based exercises and conventional PT and group B received only conventional PT. The shoulder ROM, MMT, NPRS, DASH, and SPADI were used as outcome measures. After 8 weeks of interventions, pre and post-test scores were compared, and statistical analysis was obtained. Group A showed statistically significant improvements in ROM, MMT, DASH, and SPADI scores than group B. However, in terms of pain outcome, the two groups did not show significant differences. This study has several limitations including a small sample size, and the non-blinding of the participants and therapists due to the same study set-up for both the groups. However, using a simple random sampling technique (concealed envelope) for randomization reduces the extent of bias. 

Similar to our study, Feng et al. compared the effects of VR rehabilitation and usual PT on the performance of balance and gait of Parkinson’s patients. The study consisted of two groups, one receiving VR therapy and the other receiving usual PT intervention. There were considerable improvements in balance and gait in both groups [9]. Another study showed significant improvements in upper extremity functions and strength following the administration of VR-based interventions in adjunct to conventional physical therapy in patients with chronic stroke [14]. A study used Reinforced Feedback in the Virtual Environment (RFVE) therapies for the administration of upper limb exercises in stroke individuals. In RFVE, patients perform various upper limb motor activities while holding a real object in their hands and interacting with the simulated environment. A motion-tracking device tracks the movements of the upper limb and provides feedback to the patients [15]. The findings of our study show significant improvements in DASH and SPADI scores after eight weeks of VR plus conventional physical therapy interventions. It concludes that VR-based exercises improve upper limb function and are helpful in the rehabilitation of patients with PHFs managed operatively.

A study on the effect of immersive VR on upper limb rehabilitation compared the use of hand and controller for interaction with the simulated environment in Oculus Quest. This study stated that both types of interaction help perform exercises. However, patients reported that the use of hand gestures allows the interaction in more natural and easier ways. They used three interventions for this study: lifting and holding the affected arm at a specified height, firmly gripping the affected hand, and bringing the affected hand close to the mouth [16]. On the same line, we used the games that facilitate the use of upper limbs. Reaching out for the objects at different heights, eating simulation, and opening and closing the fist reduce the ROM exercise of the affected shoulder, as well as elbow and hand grip strengthening.

However, there is limited evidence of VR's therapeutic efficacy on individuals with musculoskeletal disorders. Two research protocols were proposed to use the oculus-guided VR in combination with conventional PT for rehabilitation patients with knee osteoarthritis and distal radius fracture. The participants would be allocated into two groups, one of which would receive VR-based rehabilitation plus conventional therapy, and the other would receive only traditional therapy [12,13]. The use of VR games as a mode of training improves concentration, gives temporary relief from pain and thus facilitates the active participation of patients. A study developed three-dimensional simulated games to facilitate upper limb training for patients with shoulder impairments [5]. Our study aimed at finding the effect of VR-based interventions in adjunct to conventional PT interventions on patients with PHFs managed operatively. In this study, VR-based interventions were delivered using head-mounted oculus quests.

## Conclusions

The findings of this study reveal that virtual rehabilitation in adjunct to conventional physical therapy on proximal humerus fracture is more effective in improving shoulder ROM, muscle strength, and upper limb function than conventional therapy alone. However, no intervention can be considered superior to others in terms of the management of pain associated with proximal humerus fracture.
